# Efficacy of cardiac magnetic resonance imaging in a sub-aortic aneurysm case

**DOI:** 10.5830/CVJA-2017-027

**Published:** 2017

**Authors:** Meel Ruchika, Nethononda Richard, Peters Ferande, Essop Mohammed

**Affiliations:** Division of Cardiology, Chris Hani Baragwanath Academic Hospital and University of the Witwatersrand, Johannesburg, South Africa; Division of Cardiology, Chris Hani Baragwanath Academic Hospital and University of the Witwatersrand, Johannesburg, South Africa; Division of Cardiology, Chris Hani Baragwanath Academic Hospital and University of the Witwatersrand, Johannesburg, South Africa; Division of Cardiology, Chris Hani Baragwanath Academic Hospital and University of the Witwatersrand, Johannesburg, South Africa

**Keywords:** cardiac magnetic resonance imaging, sub-aortic aneurysm, aortic regurgitation

## Abstract

Sub-aortic (SA) aneurysms are a rare entity of variable aetiology. We report the first case of a SA aneurysm assessed using cardiac magnetic resonance imaging (MRI). A 33-year-old female with human immunodeficiency virus and on highly active antiretroviral treatment presented with syncope and dyspnoea. Clinical examination suggested moderate to severe aortic regurgitation (AR) confirmed by transthoracic and transoesophageal echocardiograms. However, echocardiography was suboptimal in defining the precise mechanism and severity of AR. A cardiac MRI was done to elucidate the aetiology, severity and mechanism of regurgitation. It confirmed the presence of a SA aneurysm below the left coronary cusp and its retraction, resulting in an eccentric AR jet. An assessment of moderate AR, based on regurgitant volume, was made. Furthermore, the anatomical relationships of the aneurysm were clearly defined. Cardiac MRI allowed comprehensive assessment of this SA aneurysm.

Sub-aortic (SA) aneurysms are a rare entity with variable aetiology. Most cases are congenital and result from a defect between the ventricular wall and valvular annuli.^1^ Earlier reports were mostly from Africa. Between 1957 and 1993, only 22 cases had been reported.^2^ Since then, isolated reports on various aspects of this rare condition have been published. There have been no reports in the literature on adult patients, using cardiac magnetic resonance imaging (MRI), to investigate SA aneurysms.

## Case report

The patient was a 33-year-old human immunodeficiency virus (HIV)-positive woman on highly active antiretroviral treatment (HAART), with a current CD4 count of 1 000 cells/µl. She was referred from a peripheral hospital, with a history of a single syncopal episode. She also admitted to a two-week history of progressive dyspnoea and fatigue (New York Heart Association functional class II). No further relevant past medical or family history was obtained.

On examination, the blood pressure was 102/52 mmHg with a pulse of 106 beats/min. No dysmorphic features were noted. She had large volume and collapsing peripheral arterial pulses, with a wide pulse pressure (50 mmHg). The apex beat was in the fifth intercostal space and displaced slightly to the left of the midclavicular line. The second heart sound was loud. There was a grade 3/4 early decrescendo diastolic murmur in the left parasternal border, characteristic of aortic regurgitation (AR). There were no peripheral stigmata of infective endocarditis. She had been treated with diuretics and there were no signs of congestive cardiac failure.

An electrocardiogram showed left ventricular (LV) hypertrophy with strain pattern in the lateral leads and left atrial (LA) enlargement. The chest X-ray was normal. The blood count was normal and the serology for syphilis and connective tissue disease was negative.

**Fig. 1:**
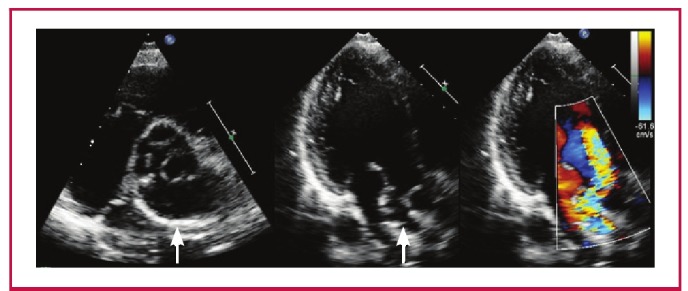
Parasternal short-axis view of the sub-aortic (SA) aneurysm (white arrow, left). Apical three-chamber views depicting the SA aneurysm (white arrow, middle), and an eccentric aortic regurgitation jet on colour flow, with flow into the SA aneurysm (right).

A transthoracic echocardiogram (TTE) revealed a dilated LV with an ejection fraction of 56% and moderate-to-severe eccentric aortic regurgitation secondary to leaflet malcoaptation (Fig. 1). There was compression of the LA by an outpouching with calcified walls adjacent to the aortic root, the exact origin and location of which was difficult to define on TTE. There was flow into and out of this structure in diastole.

A two- and three-dimensional transoesophageal echocardiogram (2D/3D TEE) was done to further define this lesion. In this patient, poor transoesophageal views prevented a full assessment of the pathology. There was a likely SA aneurysm with orifice located below the non-coronary and left coronary cusps (NCC/ LCC). The aortic leaflets and the root were normal. There was retraction of the aortic leaflets and impingement of the LA, the right pulmonary artery, and possibly the right ventricular outflow tract (RVOT) by the aneurysm. There were no associated thrombi, vegetations or other congenital lesions.

**Fig. 2:**
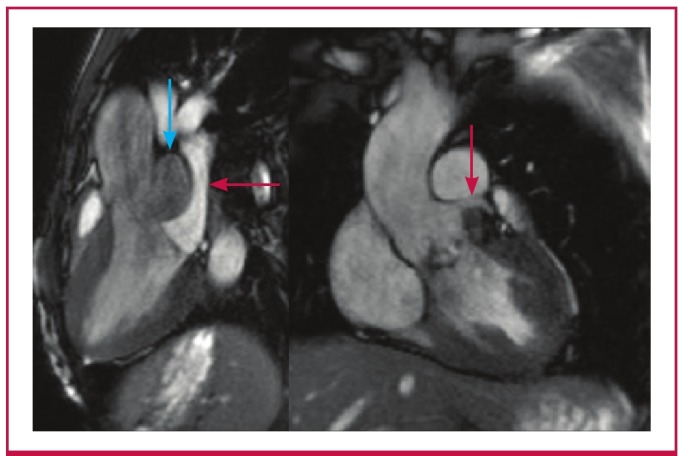
Left ventricular outflow tract views showing the subaortic aneurysm (blue arrow) compressing the left atrium (red arrow, left) and sparing the left coronary artery (right).

**Fig. 3:**
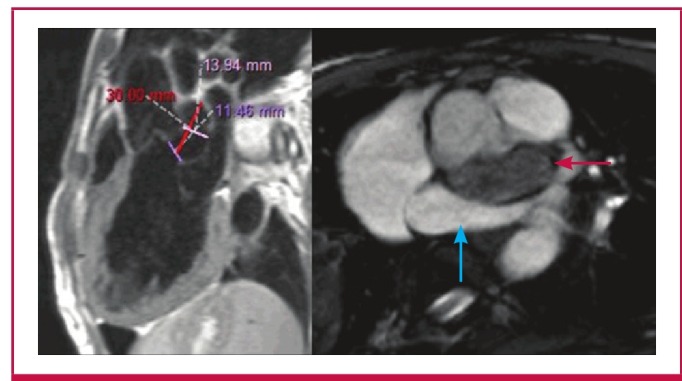
Left ventricular outflow tract (left) and short-axis views (right) depicting compression of the left atrium (blue arrow) by the sub-aortic aneurysm (red arrow).

**Fig. 4:**
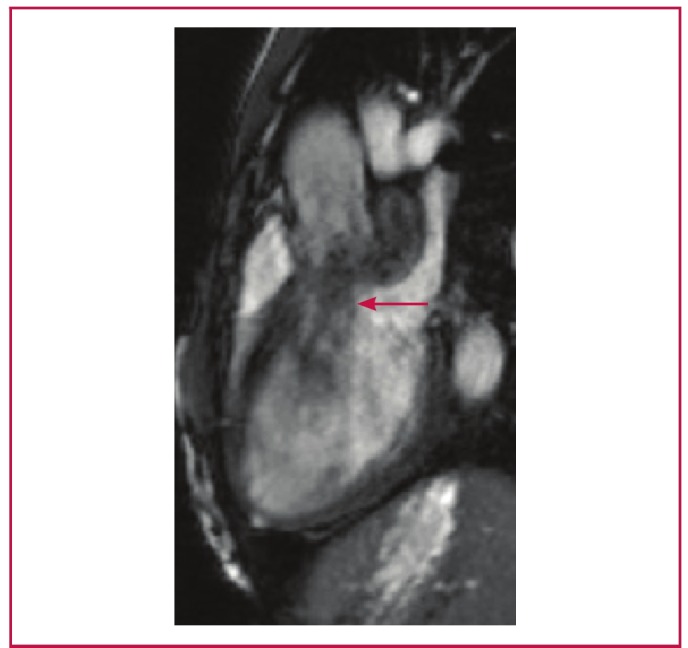
Left ventricular outflow tract MRI image showing an aortic regurgitant jet (arrow) secondary to retraction of the left coronary cusp by the sub-aortic aneurysm.

Due to suboptimal imaging on TTE and TEE, a cardiac MRI was requested to confirm the SA aneurysm and to define with certainty its relationship to the surrounding structures (Fig. 2). A 30 × 14-mm aneurysm with a 12-mm neck was noted below the aortic valve, which extended to the LA roof (Fig. 3). The communication point was just below the LCC. AR of moderate severity was noted (Fig. 4).

## Discussion

SA aneurysms are rare and postulated to be the result of a defect of congenital origin between the valvular annuli and the ventricular wall.^1^ Other possible aetiologies, although not confirmed, include tuberculosis, syphilis, rheumatic fever and infective endocarditis.^3^,^4^ Whether HIV has a causal connection also remains to be proven. These infections may merely represent an association, given their high prevalence, and causality cannot be inferred.^5^

SA aneurysms are rarer than sub-mitral aneurysms and the diagnosis is more challenging.^1^,^2^,^6^ They are mostly not suspected clinically and are found coincidentally on imaging.^7^ The chest X-ray was normal in this patient, unlike with sub-mitral aneurysms where an enlarged cardiac silhouette is often noted.^3^ SA aneurysms need to be differentiated from more commonly occurring aneurysms such as sinus of Valsalva aneurysms, which are located above the aortic valve.^6^

SA aneurysms mostly occur in young Africans.^6^ The origin is usually below the left coronary cusp, as in our patient.^2^,^6^ Clinical presentation varies, ranging from cardiac failure and systemic emboli (due to aneurysmal thrombi) to angina (due to coronary artery compression or emboli), and dysrhythmias such as ventricular tachycardia.^1^ A lack of aortic cusp support and distortion of the annulus is responsible for AR and subsequent cardiac failure.^3^

The most widely available imaging tool is TTE but this may be inadequate, as smaller aneurysms may be missed, especially in patients with suboptimal imaging windows. The diagnosis is also dependent on the skill and knowledge of the operator.^7^

Cardiac MRI is increasingly becoming a complimentary tool to echocardiography in assessing valves and congenital lesions.^8^ In patients with inadequate echocardiographic imaging, cardiac MRI allows a detailed assessment of the anatomy of congenital lesions such as SA aneurysms, and their relationship to the surrounding structures. Additionally, MRI allows accurate quantification of AR severity.

Cardiac MRI in our case allowed the precise localisation of the lesion below the LCC, which proved difficult on TTE and 2D TEE. Furthermore, involvement of the RVOT, which was suspected on 2D TEE, was excluded with certainty.

Cardiac MRI is able to accurately quantify the severity of regurgitant lesions, which may not always be possible by echocardiography alone. For example, an eccentric AR jet, as in our patient, is more accurately assessed on cardiac MRI using volumetric analysis, as opposed to less-validated echocardiographic quantification, such as the proximal isovelocity surface-area method.^8^

Two-dimensional TEE offers higher temporal resolution compared to cardiac MRI but its use is limited by angle dependence, image quality and acquisition. Cardiac MRI does not have these limitations and is able to provide improved spatial resolution.9 It also has a greater range of imaging techniques, therefore offering more anatomical and functional information. This enables enhanced clinical assessment and management.

Three-dimensional echocardiography has been used for the detection and assessment of anatomical defects in the context of congenital heart disease.^10^ It has been used to image a SA aneurysm complicating infective endocarditis.^11^ 3D TEE allowed us to define the anatomy of the SA aneurysm at the bedside but was limited by suboptimal views. The shortcomings are that it is angle dependent, the spatial and temporal resolution is still suboptimal and it is highly reliant on 2D image quality. MRI therefore proved to be an indispensable tool in this patient.

## Conclusion

Cardiac MRI is a useful adjunctive tool to echocardiography in the comprehensive assessment of SA aneurysms.
